# Developing a tool to measure satisfaction among health professionals in sub-Saharan Africa

**DOI:** 10.1186/1478-4491-11-30

**Published:** 2013-07-04

**Authors:** Adama Faye, Pierre Fournier, Idrissa Diop, Aline Philibert, Florence Morestin, Alexandre Dumont

**Affiliations:** 1 Institut de Santé et Développement, UCAD, Dakar, Senegal; 2 University of Montreal Hospital Research Centre (CRCHUM), 3875 Saint-Urbain St., 2nd Floor, Montreal, QC H2W 1V1, Canada; 3 HYGEA, Dakar, Senegal; 4 Institut National de Santé Publique du Québec, Montreal, QC, Canada; 5 Institut de Recherche Pour le Développement, UMR 216, Université Paris Descartes, Sorbonne Paris Cité, Paris, France

**Keywords:** Job satisfaction, Sub-Saharan Africa, Health workers, Measurement

## Abstract

**Background:**

In sub-Saharan Africa, lack of motivation and job dissatisfaction have been cited as causes of poor healthcare quality and outcomes. Measurement of health workers’ satisfaction adapted to sub-Saharan African working conditions and cultures is a challenge. The objective of this study was to develop a valid and reliable instrument to measure satisfaction among health professionals in the sub-Saharan African context.

**Methods:**

A survey was conducted in Senegal and Mali in 2011 among 962 care providers (doctors, midwives, nurses and technicians) practicing in 46 hospitals (capital, regional and district). The participation rate was very high: 97% (937/962). After exploratory factor analysis (EFA), construct validity was assessed through confirmatory factor analysis (CFA). The discriminant validity of our subscales was evaluated by comparing the average variance extracted (AVE) for each of the constructs with the squared interconstruct correlation (SIC), and finally for criterion validity, each subscale was tested with two hypotheses. Two dimensions of reliability were assessed: internal consistency with Cronbach’s alpha subscales and stability over time using a test-retest process.

**Results:**

Eight dimensions of satisfaction encompassing 24 items were identified and validated using a process that combined psychometric analyses and expert opinions: continuing education, salary and benefits, management style, tasks, work environment, workload, moral satisfaction and job stability. All eight dimensions demonstrated significant discriminant validity. The final model showed good performance, with a root mean square error of approximation (RMSEA) of 0.0508 (90% CI: 0.0448 to 0.0569) and a comparative fit index (CFI) of 0.9415. The concurrent criterion validity of the eight dimensions was good. Reliability was assessed based on internal consistency, which was good for all dimensions but one (moral satisfaction < 0.70). Test-retest showed satisfactory temporal stability (intra class coefficient range: 0.60 to 0.91).

**Conclusions:**

Job satisfaction is a complex construct; this study provides a multidimensional instrument whose content, construct and criterion validities were verified to ensure its suitability for the sub-Saharan African context. When using these subscales in further studies, the variability of the reliability of the subscales should be taken in to account for calculating the sample sizes. The instrument will be useful in evaluative studies which will help guide interventions aimed at improving both the quality of care and its effectiveness.

## Background

The greatest challenge facing healthcare systems in sub-Saharan Africa is the insufficiency of human resources, from both the quantitative and the qualitative standpoints. These resources, when available, tend to be concentrated in urban areas, either in the private sector or in nongovernmental organizations, which often offer better working conditions and salaries [1-3]. International migration also contributes to the shortage of health workers in sub-Saharan Africa [4]. These shortages of health professionals are a major impediment to providing good-quality care [2]. It has been estimated that sub-Saharan Africa still needs another one million or more physicians, nurses and midwives to provide the basic services required to meet the 2015 Millennium Development Goals [5]. The main consequence of this shortfall is poor quality of care and, therefore, limited impact on healthcare improvement.

Human resources availability and accessibility, while important, do not guarantee quality of care [6]. Uganda has a ratio of qualified health professionals to population three times greater than that of Bangladesh but a higher rate of maternal mortality [7]. Similar situations have been found in Russia and South Africa [7] and can be linked to health workers’ motivation [6], which has been defined as the individual’s willingness to exert and sustain the effort required to achieve the goals set by the organization [8]. Job satisfaction is a construct closely related to motivation; it is a direct result of motivational processes, of which it is the affective component [9]. Herzberg’s theory [10] distinguishes between motivating factors, which are intrinsically linked with work and determine job satisfaction, and demotivating factors, which are responsible for dissatisfaction. The consequences of providers’ satisfaction and dissatisfaction have been the subject of numerous studies. Dissatisfaction may lead to tardiness and absenteeism [11]. The inability to conduct an examination correctly, burnout, and excessive turnover have also been reported [12]. Dissatisfied providers are less courteous and tend to communicate less, or poorly, with patients. Haas *et al*. [13], in the United States, showed that patients’ sense of confidence and continuity of care were linked to physician satisfaction. In an exploratory study conducted in Taiwan, Tzeng and Ketefian [14] reported that nurses’ satisfaction was positively associated with pain management, explanations regarding care, and courtesy toward patients and their families. Studies have shown correlations between the physician-nurse relationship and mortality rates, medical errors and hospital length-of-stay [15].

Several criticisms have been expressed regarding the instruments usually employed to measure job satisfaction or motivation [16]. First, they were developed many years ago, so they may not necessarily be applicable to the new generation of health workers. Then, they were most often developed among populations of Western workers and are therefore difficult to apply to other populations in low-income countries. They fail to take into account certain dimensions of satisfaction that may be specific to a country, continent or culture, such as the value of work, social organization and religion, thereby compromising their validity.

Several studies have shown that in low-income countries, where social organization is less individualistic, intrinsic job characteristics such as challenge, recognition, autonomy and the work itself were less associated with job satisfaction, whereas extrinsic characteristics such as salary, job security and working conditions were very strongly associated with job satisfaction [17]. Several hypotheses have been advanced to explain these differences. In a study conducted in 65 countries that account for nearly 80% of the global population, Inglehart [18] suggested that these differences were due to cultural, social and political values. In another study conducted in 49 countries on five continents [17], similar results were obtained by Huang and Van De Vliert, who studied the links between job satisfaction and certain national characteristics such as the existence of social security, the country’s wealth, its degree of individualistic culture, and the distance between workers’ homes and jobs. Roe *et al*. [19] developed and tested a model on job motivation in Hungary and Bulgaria, countries that experienced a communist regime and a significant economic crisis, and compared them with the Netherlands using the same instrument. The analysis showed that the model could not easily be applied to the combined samples; it would have been better to develop a model for each country. In wealthy countries, where the basic and security-related needs that make up the first levels of Maslow’s pyramid are met, individuals are more focused on needs related to self-esteem and personal accomplishment, in contrast to people in developing countries. Social organization also plays an important role in needs satisfaction. The value attributed to self-esteem and autonomy varies depending on whether the culture of the society in which the person lives is individualistic or collectivistic. In the social hierarchies of certain societies in India or Africa, some groups at the bottom of the social ladder accord less importance to certain intrinsic work characteristics such as autonomy or advancement.

The effect of religion on job satisfaction has been mentioned [20]. Although only a few studies have looked at this relationship, some theories have been developed to attempt to explain it. In Holland’s [21] model of personality, vocational choices and work environments, individuals can be classified according to six traits. The more the work environment is aligned with a person’s trait, the greater the person’s job satisfaction. For instance, the more a person’s work environment or type of work is aligned with his religious aspirations, the more satisfied we might expect him to be. Another explanation might be found in Wilensky’s horizontal spillover theory [22], which suggests that satisfaction in one area of life spills over into a neighboring area. Thus, satisfaction in one’s spiritual life would be positively related to job satisfaction. Working in the healthcare sector is also distinctive, in that it exposes workers not only to the birth and death of individuals, but also to pain and trauma.

Studies in sub-Saharan Africa have been primarily qualitative and have generally emphasized the determinants of motivation [23]. This field of research has focused on the impact of remuneration on providers’ performance. This is explained by the fact that low salaries in the public sector have pushed many health workers to the private sector to improve their living conditions. Thus, salary increases and bonuses have become a key strategy adopted by political authorities to retain healthcare providers and improve performance. However, there is every reason to believe that, while money is necessary, its effect is not linear. Thus, the issue of what really motivates care providers in Africa has again become a core question for research on health system performance in sub-Saharan Africa. Studies by Mathauer and Imhoff in Benin and Kenya [24], Agyepong *et al*. in Ghana [25] and Dieleman *et al*. in Mali [26] have highlighted other factors such as training, supervision, and work context. There is therefore a need for a valid and reliable tool adapted to the sub-Saharan African context that would not only measure job satisfaction, but also identify its determinants and consequences. Some authors, such as Nabirye *et al*. [27], used instruments that had been previously developed among Asian populations to study the performance of nurses in Uganda. Others developed new instruments. Pillay, working in South Africa with 58 items, identified 13 factors with good internal consistency [28]. In Kenya, Mbindyo *et al*., from 23 items developed an instrument with three dimensions and 10 items [29]. However, none of these studies was involved in a process of long-term development of an instrument whose reliability and validity could be verified by a variety of procedures.

The objective of this study was to develop a valid and reliable instrument to measure the satisfaction of care providers that would be suitable for use in sub-Saharan Africa and perhaps other low-income countries. Although this posed methodological and conceptual challenges, the decision was taken to develop an instrument that could be applied to several professions and in different countries.

### Development of the instrument

**Figure 1:**
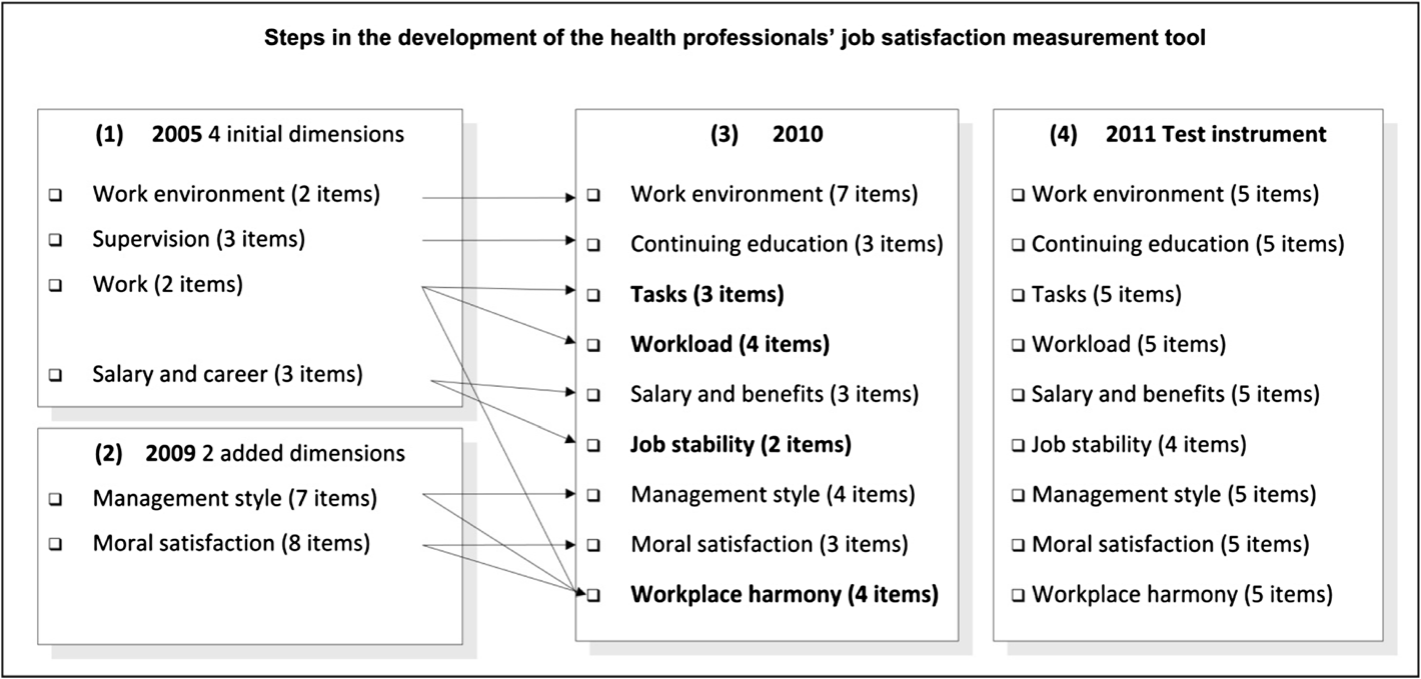
Steps in the development of the health professionals’ job satisfaction measurement tool.

Figure 1 illustrates the four stages of development of this instrument to measure health professionals’ job satisfaction. The overall purpose of this developmental process was to be able to measure adequately job satisfaction as a determinant of quality of care and of other behavioral outcomes (burnout, turnover, and so on) that undermine health system efficiency in sub-Saharan African countries. Additional information on these different development stages can be found in Additional files 1, 2 and 3.

#### Stage 1

In 2005, the first version of this instrument included four dimensions and 10 items. The four dimensions were supervision, work, work environment, and salary and career [30]. This instrument, which was based on the Measure of Job Satisfaction (MJS) [31] and the Job Descriptive Index (JDI) [32], was used as part of a preliminary study in planning for an international cooperation project.

#### Stage 2

In 2009, following a comprehensive literature review and consultations with experts (African health policy-makers, managers and practitioners) in Senegal and Mali, one dimension was changed (from supervision to continuing education) and two dimensions were added: management style [33] and moral satisfaction [34]. The number of items to be tested grew to 42.

#### Stage 3

This instrument was tested with 899 health professionals in Senegal in 2008 and in Mali in 2010. Following a series of factor analyses and consultations with experts, the structure of the measurement instrument was modified. Three new dimensions were added to the six initial ones (33 items). Two of these resulted from subdividing two existing dimensions (1: *work organization* was subdivided into *tasks* and *workload*, and 2: *salary and benefits* was subdivided into *salary and benefits* and *job stability*); the third new dimension added was *workplace harmony*, which emerged from items arising out of *moral satisfaction*, *management* and *organization.* From the construct standpoint, this instrument was seen to be valid because, on one hand, seven of its dimensions are among the 11 that van Saane *et al*. identified in an exhaustive review of job satisfaction measurement instruments [34], and those authors considered that a valid instrument should have 7 of the 11 dimensions. On the other hand, the two remaining dimensions were appropriate to the cultural context and to low-income countries. These were *job stability* and *work environment,* extrinsic characteristics that were found by several authors to be important components of job satisfaction among healthcare workers in Africa [33]. Job stability appeared also to be important because of the growing trend in these countries over the past decade or more to hire health personnel on a contractual basis rather than as permanently appointed civil servants, as was previously the case. This dual perspective, universal and contextual, was appropriate within the framework of the approach we adopted.

#### Stage 4

The instrument tested in this study, which is the fourth and final stage of development, included nine dimensions and 44 items.

## Materials and methods

### Study population and sample

The target population consisted of doctors, nurses and midwives working in referral health facilities in sub-Saharan Africa. The final stage, conducted in Senegal and Mali, involved the health professionals working in the 46 hospitals selected for the QUARITÉ trial, conducted between 2007 and 2011 [35]. The objective of that randomized trial was to evaluate the effectiveness of a series of strategies to reduce maternal mortality in referral health facilities. That trial included a component to analyze satisfaction among health professionals, which was one of the intermediary mechanisms expected from the effects produced by the intervention. The trial was conducted in 46 out of a total of 49 eligible referral hospitals - 23 in Mali and 26 in Senegal - spread across both countries. A hospital was eligible for the trial if it had functional operating rooms and carried out more than 800 deliveries annually. Three eligible hospitals were excluded: two already had a structured program for carrying out maternal death audits before the project began, and for one other hospital, written consent was not provided by local authorities. The 46 included hospitals are representative of the existing health system in Senegal and Mali, taking into account the variety of the contexts (urban versus rural) and of the levels of care (primary versus secondary referral health facilities).

For the purposes of this survey, the sample consisted of all physicians, nurses, midwives and health technicians practicing in the inpatient maternity wards of the 46 health facilities in the QUARITÉ trial (962 persons).

### Data collection

Data were collected from January to June 2011 using a questionnaire administered by surveyors during interviews with individuals at their workplace; in cases where respondents were not on their work site, up to two recalls were done. The data were collected in each country by two surveyors who had previously participated in data collections for the QUARITÉ trial, were very experienced and had good knowledge of the questionnaire. All the surveyors were trained by the same experts, which helped to standardize the instrument.

The questionnaire contained one question for each of the 44 items of satisfaction. These were Likert-type questions based on a five-point scale (very satisfied, satisfied, somewhat satisfied, dissatisfied, very dissatisfied). It also collected information on personal characteristics such as sex, age, professional qualifications, family structure and union affiliation. (see Additional file 4)

### Exploration

First, an exploratory factor analysis (EFA) was used as a variable reduction technique to identify the number of latent constructs and the underlying factor structure that account for the covariation among our 44 items. The extraction method used was principal component analysis based on a correlation matrix. The model was optimized through an orthogonal rotation (Varimax with Kaiser normalization). The extraction rules we used for our 44 items were the following ones: an extraction value equal or higher to 0.5, a loading factor on a first factor of 0.5 or higher and on a second factor equal or lower than 0.35.

### Psychometric validation

1. Validity

1.1 Construct validity

As a theory-testing model, a confirmatory factor analysis (CFA) was performed to test and validate the factor structure of our latent constructs [36]. The model resulting from EFA was tested as an oblique model given since the eight factors were correlated between each other. The estimation procedure used was the maximum likelihood based on an input variance-covariance matrix. The fit indices we used were the comparative fit index (CFI) [37] and the root mean square error of approximation (RMSEA) [38]. Robust estimates of standard errors were also evaluated (Satorra-Bentler).

1.1 Discriminant validity

The discriminant validity between the different dimensions is demonstrated if the average variance extracted (AVE) estimates are greater than the corresponding squared interconstruct correlation (SIC) estimates, and if the correlation coefficient between any two dimensions is less than one minus two times the standard error of their correlation coefficient [39].

1.1 Criterion-related validity

Assessment of concurrent criterion-related validity consisted of verifying whether test scores of the different dimensions were correlated with criteria measured at the same time [40]. Validity criterion is usually based on comparison between an existing scale and the one under development. In our case, as such appropriate scales do not exist for our dimensions, for each of them we selected two criteria based on a hypothesis suggested by an expert’s panel. Tso *et al*. used the same approach for criterion-related validation of their patient satisfaction scale; they correlated the patient satisfaction scores with hypothetically related criteria (intended future reutilization and recommendation to others) [41]. For each dimension, two hypotheses were formulated regarding the links between their scores and characteristics of respondents or of their work setting. The dimension score was obtained by adding the item scores, weighted by their loading on their dimension. The score was divided into quintiles, with the top quintile considered as ‘very satisfied’. Chi-squared testing was used for comparisons. Data used to test the hypotheses came either from the questionnaire administered in the surveys (characteristics related to sociodemographics, work and profession) or from other sources of data collected during the QUARITÉ trial.

2. Reliability

2. 1 Internal consistency

The internal consistency of each component of the instrument was assessed using Cronbach’s alpha. For a component to be considered consistent, the value of the coefficient had to be above 0.70 [34]. We sought to improve consistency by removing items one at a time and recalculating the coefficient.

2. 1 Stability

Stability over time was explored using a test-retest process [42]. The questionnaire was administered and then re-administered 15 days later to 25 subjects working in institutions that were not part of the QUARITÉ trial [35]. The correlation between two measures for the same professional, that is, the reproducibility of the questionnaire, was assessed for each dimension based on the intra class correlation coefficient (ICC). Fleiss considers that an ICC value from 0.40 to 0.75 as fair to good [43].

Statistical analyses were performed with SPSS Inc. Released 2010. SPSS Statistics for Windows, Version 19.0. Chicago: SPSS Inc. and R v2.14.1 (Foundation for Statistical Computing, Vienna, Austria. ISBN 3-900051-07-0, URL http://www.R-project.org/).

### Ethical considerations

The QUARITÉ project was approved by the Research Ethics Committee of Sainte-Justine Hospital Center (protocol number 2425), as well as by the National Ethics Committees of Senegal and Mali, in 2006. For this survey, informed consent was first obtained from each person interviewed. Data were processed anonymously.

## Results

### Characteristics of the health professionals surveyed

The sample consisted of 962 professionals working in the health facilities that were part of the QUARITÉ project; of these, 937 were interviewed (participation rate 97.4%). The respondents’ average age was 40.3 years (SD = 9.4), and 69% of them were women. Approximately half of them (46%) worked in the capitals (Bamako and Dakar).

The sample was randomly divided into two subsamples. To ensure better comparability, they were stratified by country and profession. EFA was conducted on the first subsample and CFA on the second one. Additional file 5 shows items scores (mean, standard deviation, median) for the two sub samples and the total sample (Table 1).

**Table 1:** Sample and sub sample characteristics

	**Sub sample 1**	**Sub sample 2**	**Total**
	**N (%)**	**N (%)**	**N (%)**
Country			
Senegal	221 (47%)	222 (47%)	443 (47%)
Mali	247 (53%)	247 (53%)	494 (53%)
Type of facility			
Capital	217 (46%)	218 (47%)	435 (46%)
Regional	139 (30%)	130 (28%)	269 (29%)
District	112 (24%)	121 (26%)	233 (25%)
Age (years/months)	40/11	39/8	40/4
Male	147 (31%)	142 (30%)	289 (31%)
Female	321 (69%)	327 (70%)	648 (69%)
Profession			
Physician	92 (20%)	93 (20%)	185 (20%)
Midwife	208 (44%)	210 (45%)	418 (45%)
Nurse	89 (19%)	88 (19%)	177 (19%)
Health technician	79 (17%)	78 (17%)	157 (17%)

### Exploration

Following the EFA, eight factors were retained: all of them had an eigenvalue greater than 1 (decreasing from 6.255 to 1.113) with a scree plot showing a discernible elbow from the eighth factor (ninth factor eigenvalue = .685). Among the 44 initial items, 24 of them met the inclusion criteria (see loading coefficients in Table 2 and the component matrix in Additional file 6). Percentage of total variance explained by the eight factors was 72.8% (Table 2). A Kaiser-Meyer-Olkin measure of sampling adequacy higher than .5 (.875) and a statistically significant Bartlett sphericity test (.000) insured the appropriateness of the analysis.

**Table 2:** Satisfaction scale - dimensions and items

**Dimensions**	**Items**	**Loading coefficient**	**Communality**	**Variance (cumulative)**	**α**^**a**^
F1 Continuing	(Q28) relevance	0.856	0.784	0.148	0.90
education	(Q30) skills acquired	0.851	0.791	(0.148)	
	(Q29) skills utilization	0.810	0.723		
	(Q26) continuing education you still receive	0.779	0.706		
	(Q27) selection for training	0.722	0.703		
F2 Tasks	(Q19) job description	0.856	0.829	0.095	0.81
	(Q20) job description and effective tasks	0.825	0.819	(0.243)	
	(Q18) level of responsibility	0.746	0.631		
F3 Salary and	(Q1) level of salary	0.856	0.752	0.094	0.83
benefits	(Q3) salary and needs	0.827	0.715	(0.336)	
	(Q4) level of salary and workload	0.819	0.712		
F4 Workload	(Q12) workload	0.858	0.789	0.092	0.77
	(Q11) work schedule	0.815	0.713	(0.428)	
	(Q14) balance between care and other activities	0.709	0.605		
F5 Management style	(Q33) information about your department	0.835	0.764	0.087	0.83
	(Q32) participation in decision making	0.751	0.685	(0.515)	
	(Q34) information about your institution	0.721	0.667		
F6 Job stability	(Q42) concern about losing your job	0.843	0.734	0.084	0.73
	(Q41) salary paid on time	0.807	0.682	(0.599)	
	(Q44) status (civil servant, tenure track, contract)	0.726	0.598		
F7 Work environment	(Q8) availability of equipment and materials	0.856	0.781	0.065	0.77
	(Q7) availability of medicines	0.831	0.762	(0.664)	
F8 Moral satisfaction	(Q38) support to patients from a religious point of view	0.851	0.764	0.064	0.68
	(Q37) quality of your work	0.829	0.772	(0.728)	

^a^Cronbach’s α. Cronbach’s α for the 24 items = 0.794.

### Validity

#### Construct validity

Confirmatory factor analysis was carried out on the eight dimensions and 24 items retained at the end of the exploratory phase. The results showed good model fit, with a CFI of 0.9415 and a RMSEA = 0.0508 (90% CI: 0.0448 to 0.0569).

#### Discriminant validity

All average variance extracted (AVE) estimates were greater than the corresponding squared interconstruct correlation (SIC) estimates and the correlation coefficient is less than one minus two times the standard error of their correlation coefficient. This means the indicators had more in common with the construct with which they were associated than they did with other constructs. Therefore, our model showed good discriminant validity (Table 3).

**Table 3:** Discriminant validity: average variance extracted estimates (AVE) and squared interconstruct correlation estimates (SIC)

	**F1**^**a**^	**F2**	**F3**	**F4**	**F5**	**F6**	**F7**	**F8**
AVE	0.648	0.657	0.696	0.634	0.594	0.630	0.712	0.706
SIC								
F1	.	0.140	0.029	0.065	0.213	0.049	0.053	0.055
F2		.	0.208	0.375	0.381	0.108	0.244	0.300
F3			.	0.203	0.210	0.159	0.215	0.083
F4				.	0.290	0.200	0.285	0.224
F5					.	0.191	0.236	0.199
F6						.	0.124	0.185
F7							.	0.035

^a^F1: continuing education; F2: tasks; F3: salary and benefits; F4: workload;

F5: management style; F6: job stability; F7: work environment; F8: moral satisfaction.

#### Criterion-related validity

The hypotheses (two per dimension) were verified for seven dimensions, for the eighth (tasks) only one was verified (satisfaction higher in lower level facilities as they are more diversified). For the second one (satisfaction higher for managers) the association was on the hypothesized direction but not statistically significant (*P* = 0.07) (Table 4).

**Table 4:** Criterion validity - dimensions and hypotheses

		**n**	**% very satisfied**^**a**^	***P *(χ**^**2**^**)**
F1 Continuing education				
1. Management position	yes	357	23.8	0.01
	no	580	17.2	
2. Tenure track position	yes	722	22.2	0.00
	no	215	11.7	
F2 Tasks				
1. Facility level	national	435	11.5	0.00
	regional	285	16.1	
	district	217	22.6	
2. Management position	yes	357	13.4	0.07
	no	580	9.7	
F3 Salary and benefits				
1. Facility level	national	435	11.5	0.00
	regional	285	16.1	
	district	217	22.6	
2. Dependents	low	407	12.5	0.00
	high	526	18.3	
F4 Workload				
1. Deliveries per midwife	low	175	20.0	0.03
	high	762	13.1	
2. Married	yes	777	20.4	0.02
	no	160	11.3	
F5 Management Style				
1. Management position	yes	357	22.7	0.00
	no	580	13.8	
2. Facility level	national	435	15.4	0.00
	regional	285	14.0	
	district	215	24.9	
F6 Stability				
1. Tenure track position	yes	694	19.5	0.00
	no	242	6.2	
2. Facility level	national	435	15.4	0.02
	regional	285	12.6	
	district	215	24.9	
F7 Work environment				
1. Equipment for deliveries	good	128	40.6	0.00
	poor	809	24.7	
2. Equipment for postpartum care	good	222	40.0	0.02
	poor	640	25.8	
	district	215	21.7	
F8 Moral satisfaction				
1. Sex	male	289	23.9	0.00
	female	648	34.6	
2. Tenure track position	yes	694	20.5	0.00
	no	242	11.4	

^a^highest quintile scores.

#### Reliability

##### Internal consistency

Cronbach’s alpha coefficient was above 0.70 for all factors except moral satisfaction (0.68).

##### Stability

Three of the components were on the upper part of the ‘fair to good’ range (moral satisfaction, work environment, management) and five over that range (stability, tasks, continuing education, workload, salary and benefits). Globally the temporal stability was good (see Table 5).

**Table 5:** Satisfaction dimensions: scores and test-retest

**Dimensions**	**ICC**^**a**^	**Mean**^**b**^	**SD**^**b**^
Moral satisfaction	0.67	77.77	12.39
Stability	0.81	71.96	17.62
Tasks	0.82	67.37	15.98
Workload	0.76	58.26	18.12
Work environment	0.60	53.49	20.38
Continuing education	0.86	53.00	21.89
Management	0.67	48.00	19.36
Salary and benefits	0.91	38.47	17.56

^a^intraclass coefficient.

^b^standardized (on 100).

## Discussion

The dimensions found in our study are consistent with studies on satisfaction carried out in developing countries in general, and in sub-Saharan Africa in particular, but have never before been together in a single multifaceted instrument. They include dimensions that have been explored in many studies, such as salary, management, continuing education, work environment, and evaluation of work by one’s peers and superiors [33]. This instrument has the added advantage of exploring dimensions not well documented such as moral satisfaction and job stability. Particular attention should be given to these dimensions in future developments for a better grasp of all dimensions of satisfaction in developing countries and in sub-Saharan Africa.

While there have been several attempts to develop instruments to measure job satisfaction in Africa, the analysis of their validity and reliability has generally been limited. Most of the studies, because of their qualitative nature [24,26,44-48], or even if they were quantitative [24,25,27,49,50], primarily explored content validity. The study of their construct validity was essentially limited to multidimensional exploration (factor or principal component analysis) [28,29,51]. In this study, in addition to doing confirmatory factor analysis, we studied criterion validity and discriminant validity to better assess the constructs [40]. Instruments developed in Africa have also suffered from a lack of attention to reliability. In this study, we analyzed two principal components of reliability, which are, internal consistency and temporal reliability [34,40].

The iterative process used to develop this instrument combined a variety of surveys and their psychometric analyses, literature reviews, and expert consultations in order to ensure the specific features of the sub-Saharan African context were taken into account. This instrument’s content validity and construct validity are both very satisfactory. Confirmatory factor analysis, with an RMSEA of 0.94 and a CFI of 0.0508, showed good model fit [52].

This instrument underwent concurrent criterion-related validation. All hypotheses were validated excepted one, a noteworthy result. The results of these tests showed, incidentally, that managers derive more benefits from their positions, leading to greater satisfaction in terms of continuing education and management style. Similar results have been obtained in other studies in Africa [32] and other developing countries [9]. Women were more satisfied than men with the work they produced and with their relationships with colleagues. Another observation was that smaller facility size was associated with greater staff satisfaction with salary, tasks and management.

A longitudinal study of this research program, using an earlier version of this instrument applied to midwives in Senegal, showed that several of its dimensions also had good long term predictive validity: dissatisfaction with salary and work tasks was associated with emotional exhaustion (one of the three dimensions of burnout), and dissatisfaction with continuing education was a predictor of intention to quit [12].

Globally temporal stability was good, but as Fleiss mentions, as reliability affects the power of statistical tests (the lower the reliability, the greater the risk of type two error) sample size should be adjusted accordingly [43].

Internal consistency varied for the different dimensions and was above 0.7 for seven of the eight dimensions. The Cronbach’s alpha value for moral satisfaction was 0.68, which limits the usefulness of this dimension and calls for caution in its interpretation and further utilization.

The results showed marked variation in levels of satisfaction from one dimension to another. The providers were much less satisfied with their salaries (38.47/100), management (48.00/100) continuing education (53.00/100) and work environment (53.49/100). In a review of the literature on studies conducted between 1980 and 2007, Willis-Shattuck *et al*. [33] showed that pay was the most important factor in staff motivation and retention in developing countries. Similar results were obtained by Agyepong *et al*. [25] in Ghana, Leshabari *et al*. [53] and Chandler *et al*. [51] in Tanzania, Pillay [28] in South Africa, Dieleman *et al*. [26] in Mali, and Ndiaye *et al*. [50] in Senegal. Non-financial factors are also important [24]. The availability of appropriate tools and supports for their work is a key factor of satisfaction [25,48,53], especially in rural areas [54,55] and in places where the minimal conditions required for work are not present [23,45,49]. The management style in Africa is often disparaged and is a source of dissatisfaction, with the main reasons being favoritism and a lack of consultation in decision-making [24]. The basis upon which people are selected for training need to be transparent, otherwise those selections create frustrations [23,26] and lead to job dissatisfaction [23]. There are definitely opportunities in this area for improvements to working conditions that would have a positive impact not only on job satisfaction among healthcare workers, but ultimately also on the care they provide.

Workers were more satisfied with their professional accomplishments (moral satisfaction: 77.77/100). The results obtained in this regard were not unequivocal. The need for recognition by one’s colleagues and superiors is a strong motivational factor, particularly in low-income countries. A good physician-nurse relationship reduces stress, anger and frustration on both sides and is an important factor in job satisfaction [56,57]. However, some studies found providers to be dissatisfied especially in their relationships with their superiors, due to a lack of respect [47]. This was also true with regard to moral satisfaction. Providers often assessed their work positively [29,48,57]. However, they were often less satisfied with the community’s assessment of their work [24]. This difference is likely due to the fact that the items measuring this dimension approached it from a variety of angles.

### Strengths and weaknesses

The key strength of this study lies in the validity of the instrument developed. The various iterations and tests ensured good content, construct and criterion validities. The surveys undertaken in this process were conducted with great care to ensure quality and data saturation. In the final survey, the participation rate was 97.4%, with data saturation of 98.6%; this helped to limit the use of procedures for imputation of missing data, which is always problematic. The sample was diversified, covering two countries and several professional groups working at different levels of the health system. Thus generalization to similar countries and several professions is improved.

The key weaknesses concerned reliability. The moral satisfaction dimension presented insufficient internal consistency (0.68) and, of the others, only four had high coefficients (Cronbach’s alpha > 0.80). The diversity of professions and work settings, combined with the fact that these procedures were carried out in two countries, resulted in an unavoidable heterogeneity that translated into some lost reliability. This was a necessary trade-off in order to produce an instrument that could be used in different work settings and with several professions. While generalization is improved due to the sample’s diversity, the fact that the respondents all worked in maternity services could limit generalization to all health sectors. Given the mobility of health workers, with the exception of midwives, from one sector to another one, this limitation might not be considered a major constraint.

For further utilization of this tool and its subscales, the variability of their reliability should be taken in account and the sample sizes increased in order to take in account the increased risk of type two error.

## Conclusions

Following a long process of development, the instrument to measure satisfaction among health professionals in sub-Saharan Africa presents strong validity. Its reliability could be improved. The most important dimensions for measuring professional satisfaction in Africa have been identified and validated. With some adjustments, this instrument can be used in very different settings, as well as with different professional groups. Other studies in different sub-Saharan African countries will help to get a better understanding of this complex phenomenon.

This instrument, with its future developments and applications in the field, will be useful in contributing to the development of a corpus of knowledge on job satisfaction in sub-Saharan Africa and other low-income countries. This will help to address a major issue, since although job satisfaction is known to be problematic and dissatisfaction can have many negative consequences, there is currently not enough knowledge available to measure the progress of improvements that are implemented or to assess their impacts.

## Abbreviations

AVE: Average variance extracted; CFA: Confirmatory factor analysis; EFA: Exploratory factor analysis; CFI: Comparative fit index; ICC: Intra class coefficient; RMSEA: Root mean square error of approximation; SD: Standard deviation; SIC: Squarred interconstruct correlation.

## Competing interest

The authors declared that they have no competing interest.

## Authors’ contributions

AF wrote the first draft of the manuscript with PF and undertook the statistical analysis with AP. PF led the development of the study from its inception. ID contributed as a field expert at several stages of the instrument’s development and supervised all the data collection in Senegal. AP designed the psychometric analysis. FM was responsible for implementing the changes in the measurement instrument and participated in the data collection in Senegal. AD participated in the development process since 2009. All authors read, improved and approved the final manuscript.

## Supplementary Material

Additional file 1 Stage 1. Click here for file

Additional file 2 Stage 2. Click here for file

Additional file 3 Stage 3. Click here for file

Additional file 4 Questionnaire - Satisfaction professionnelle (deuxième passage). Click here for file

Additional file 5 Items (mean, standard deviation, median)*. Click here for file

Additional file 6 Component matrix. Click here for file
