# Supplementary material for: Developing a tool to measure satisfaction among health professionals in sub-Saharan Africa
Source: Hum Resour Health. 2013 Jul 4;11:30. doi: 10.1186/1478-4491-11-30 (PMC3704923; doi:10.1186/1478-4491-11-30)
Supplement: Additional file 1 — Stage 1. [file 1478-4491-11-30-S1.docx]

Additional file 1: Stage 1

N = 333 (doctors, nurses, assistant nurse, non medical staff)

Settings: Mali (Kayes, Ségou)

Primary health care facilities

Data collection: March to May 2002

| **Dimensions and items** | Loading coefficient | Variance  (cumulative) | Cronbach  α |  |
| --- | --- | --- | --- | --- |
| **Salary and career** |  |  |  |  |
| Bonuses | -0.890 | 0,31  (0,31) | 0,81 |  |
| Salary | -0.876 |  |  |  |
| Career and promotion | -0.777 |  |  |  |
| **Supervision** |  |  |  |  |
| Supervision contents | 0.917 | 0,19  (0,50) | 0,76 |  |
| Supervision frequency | 0.904 |  |  |  |
| Information received about the centre | 0.561 |  |  |  |
| **Work** |  |  |  |  |
| Work schedule | 0.863 | 0,12  (0,62) | 0,43 |  |
| Workload | 0.744 |  |  |  |
| **Work environment** |  |  |  |  |
| Medical equipment | 0.895 | 0,11  (0,73) | 0,55 |  |
| Documents (guidelines/protocols) | 0.819 |  |  |  |
| Items removed before analysis (missing data>30%) | | | | |

- Benefits in kind
- Initial training
- Continuing education
- Relationships with colleagues
- Involvement in center management
- Drugs

Reference:

Dufresne Caroline. Réforme des systèmes de santé et satisfaction du personnel ‘Cas du Mali’ Mémoire de Maîtrise, Administration de la santé, Université de Montréal, 2005.
