# Supplementary material for: Developing a tool to measure satisfaction among health professionals in sub-Saharan Africa
Source: Hum Resour Health. 2013 Jul 4;11:30. doi: 10.1186/1478-4491-11-30 (PMC3704923; doi:10.1186/1478-4491-11-30)
Supplement: Additional file 2 — Stage 2. [file 1478-4491-11-30-S2.docx]

**Additional file 2: Stage II**

After a literature review and experts consultations new dimensions and items were added or reintroduced after stage 1 (in italics):

| **Salary and benefits (6)**  Q1 Salary  *Q2 Salary paid on time*  Q3 Bonuses  *Q4 Benefits in kind*  Q5 Career and promotion  *Q6 Concern about losing your job* | ***Continuing education (4)***  *Q24 Initial training and actual tasks*  *Q25 Continuing education you still receive*  *Q26 Selection for training*  *Q27 Support from supervisors* |
| --- | --- |
| **Work environment (8)**  Q 7 Medical equipment  *Q8 Premises*  *Q9 Blood for transfusion*  *Q10 Drugs*  *Q11 Consumables(cotton, alcohol)*  *Q12 Protection against professional risks*  *Q13 Documents (administrative)*  Q14 Documents (guidelines/protocols) | **Moral satisfaction (8)**  *Q28 Population opinion about your institution*  *Q29 Quality of care for patients*  *Q30 Involvement in deliveries for mothers and babies*  *Q31 Quality of your work*  *Q32 Service provided to patients*  *Q33 Acknowledgment of your work by patients*  *Q34 Acknowledgment of your work by colleagues*  *Q35 Acknowledgment of your work by superiors* |
| **Work organization (9)**  Q15 Work schedule  Q16 Workload  *Q17 Distribution of workload among co-workers*  *Q18 Sense of rapports among co-workers*  *Q19 Diversity of tasks*  *Q20 Balance between clerical tasks and care*  *Q21 Fit between your tasks and your skills*  *Q22 Level of responsibility*  *Q23 Collaboration with other hospital departments* | **Management style (7)**  *Q37 Rewards policy*  *Q36 Penalties policy*  *Q38 Evaluation process*  *Q39 Respect from your superiors*  *Q40 Participation in decision making*  *Q41 Information about your department*  *Q42 Information about your institution* |
