# Supplementary material for: Developing a tool to measure satisfaction among health professionals in sub-Saharan Africa
Source: Hum Resour Health. 2013 Jul 4;11:30. doi: 10.1186/1478-4491-11-30 (PMC3704923; doi:10.1186/1478-4491-11-30)
Supplement: Additional file 3 — Stage 3. [file 1478-4491-11-30-S3.doc]

**Additional file 3 Stage 3**

N = 899 (doctors, midwifes, nurses, technicians)

Settings: Mali and Senegal

Quarite facilities (National, Regional and District Hospitals)

Data collection: January to June 2008 (Senegal) and February to June 2010 (Mali)

| **Dimension** | **Items** | **Loading coefficient** | **Variance**  **(cumulative)** | **Cronbach**  **α** |
| --- | --- | --- | --- | --- |
| **Work environment** | Q7 Medical equipment | 0.717 | 11.18 | 0.83 |
|  | Q8 Premises | 0.555 | (11.18) |  |
|  | Q9 Blood for transfusion | 0.558 |  |  |
|  | Q10 Drugs | 0.727 |  |  |
|  | Q11 Consumables(cotton, alcohol) | 0.762 |  |  |
|  | Q12 Protection against professional risks | 0.623 |  |  |
|  | Q13 Documents (administrative) | 0.671 |  |  |
| **Management style** | Q37 Rewards policy | 0.595 | 8.67 | 0.78 |
|  | Q40 Participation in decision making | 0.553 | (19.85) |  |
|  | Q41 Information about your department | 0.798 |  |  |
|  | Q42 Information about your institution | 0.741 |  |  |
| **Workplace harmony** | Q18 Sense of rapports among co-workers | 0.470 | 7.23 | 0.65 |
|  | Q34 Acknowledgment of your work by colleagues | 0.689 | (27.08) |  |
|  | Q35 Acknowledgment of your work by superiors | 0.705 |  |  |
|  | Q39 Respect from your superiors | 0.636 |  |  |
| **Workload** | Q15 Work schedule | 0.758 | 7.14 | 0.75 |
|  | Q16 Workload | 0.759 | (34.22) |  |
|  | Q17 Distribution of workload among co-workers | 0.574 |  |  |
|  | Q20 Balance between clerical tasks and care | 0.603 |  |  |
| **Moral satisfaction** | Q30 Involvement in deliveries for mothers and babies | 0.668 | 6.75 | 0.63 |
|  | Q31 Quality of your work | 0.741 | (40.97) |  |
|  | Q32 Service provided to patients | 0.556 |  |  |
| **Tasks** | Q19 Diversity of tasks | 0.646 | 5.44 | 0.70 |
|  | Q21 Fit between your tasks and your skills | 0.756 | (46.41) |  |
|  | Q22 Level of responsibility | 0.702 |  |  |
| **Continuing education** | Q25 Continuing education you still receive | 0.554 | 5.29 | 0.72 |
|  | Q26 Selection for training | 0.546 | (51.70) |  |
|  | Q27 Support from supervisors | 0.462 |  |  |
| **Salary and benefits** | Q1 Salary | 0.740 | 4.51 | 0.59 |
|  | Q3 Bonuses | 0.775 | (56.21) |  |
|  | Q4 Benefits in kind | 0.610 |  |  |
| **Job stability** | Q2 Salary paid on time | 0.808 | 3.47 | 0.58 |
|  | Q6 Concern about losing your job | 0.797 | (59.68) |  |

9 items did not satisfied inclusion criterion (loading>0.35 on one dimension and not>0.25 in an another one)

Cronbach α (33 items) = 0.896
