# Supplementary material for: Developing a tool to measure satisfaction among health professionals in sub-Saharan Africa
Source: Hum Resour Health. 2013 Jul 4;11:30. doi: 10.1186/1478-4491-11-30 (PMC3704923; doi:10.1186/1478-4491-11-30)
Supplement: Additional file 4 — Questionnaire - Satisfaction professionnelle (deuxième passage). [file 1478-4491-11-30-S4.doc]

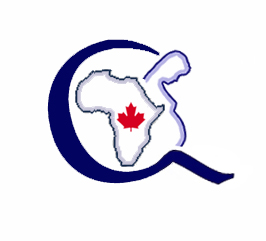
 **UARITE**

***Qualité des soins, gestion du risque et techniques obstétricales***

**Questionnaire - Satisfaction professionnelle (deuxième passage)**

| **Date : ____________________**  **Nom de l’établissement : _______________________________________**  **Nom de l’enquêteur : __________________________________________** | |
| --- | --- |
| **Code du répondant : /__/__/__/__/__/** (Rappel : anonymat) | |
| **Sexe :** 1 Masculin 2 Féminin | /____/ |
| **Profession :**  1 Gynécologue-obstétricien *(si interne ou CES : cocher ci-dessous)*  2 Médecin compétent en SONU  3 Médecin généraliste non formé en SONU  4 Pédiatre  5 Médecin anesthésiste  6 Interne  7 CES  8 Sage-femme  9 Infirmier(e) diplômé(e) / technicien(ne) sup. à la maternité  10 Infirmier(e) / technicien(ne) sup. anesthésiste  11 Instrumentiste  12 Aide opérateur | /__/ /__/ |
| **Niveau d’instruction :**  1 Non alphabétisé  2 Primaire  3 Secondaire – BFEM ou diplôme de même niveau  4 Secondaire – Baccalauréat ou diplôme de même niveau  5 Supérieur | /____/ |
| **Occupez-vous un poste de cadre ?** 1 Oui 2 Non | /____/ |
| **Si oui, lequel :**  1 Chef de service  2 Professeur d’université  3 Infirmier-chef  4 Maîtresse sage-femme  5 Chef d’unité de soins  6 Autre : __________________________________ | /____/ |
| **Âge :** ________ ans | /___//___/ |
| **Ancienneté dans le** ***poste actuel*** :_______ années | /___/ /___/ |
| **** Si la durée dans le poste actuel est* ***inférieure à 1 an****, préciser*  *s’il s’agit d’un:*   1. nouveau recrutement 2. changement de service ou d’établissement   préciser le nom de l’établissement __________________________ | /____/ |
| **Ancienneté dans la** ***profession*** :________ années | /____/ |
| **Statut Professionnel :**  1 Fonctionnaire  2 Militaire hors cadre  3 Contractuel Etat  4 Contractuel hôpital  5 Contractuel commune  6 Bénévole communautaire | /____/ |
| **Travail *pour la maternité* :** 1 À temps plein 2 À temps partiel | /____/ |
| **Affiliation syndicale :** 1 Syndiqué(e) 2 Non syndiqué(e) | /____/ |
| **Situation matrimoniale :**   1. Marié - Polygame 2. Marié – Monogame 3. Célibataire 4. Veuf (ve) 5. Divorcé(e) | /____/ |
| **Combien de personnes prenez-vous en charge avec votre salaire**[[1]](#footnote-2) **?** (en comptant vous-même, enfants, parents, domestique…) : | /___/ /___/ |
| **Habitez-vous loin de votre famille à cause de votre travail ?**  1 Oui 2 Non | /____/ |
| **Temps de trajet entre domicile et lieu de travail :**  **Aller :** _____ h _____min.  **Retour :** _____ h _____min. | /__/__/__/__/  /__/__/__/__/ |
| **Moyen de transport principal :**  1 Voiture personnelle  2 Moto, mobylette  3 Transport en commun  4 Charrette  5 Vélo  6 À pied | /____/ |

**Instructions :** Nous allons vous poser une liste de questions sur votre satisfaction dans votre travail.

Les questions portent sur différents aspects de la satisfaction : 1) la rémunération; 2) les outils et le cadre de travail; 3) la charge de travail; 4) la tâche; 5) l’entente au travail; 6) la formation continue; 7) le management; 8) la satisfaction morale et 9) la stabilité.

Pour chaque question, il y a 5 choix de réponse (Donner la feuille avec les choix de réponse). Il n’y a pas de réponse vraie ou fausse : ce qui compte c’est que vous exprimiez votre opinion.

1 Questions sur la rémunération

| 1 | Êtes-vous content(e) du montant de votre salaire ? | 5  Très content | 4  Content | 3 Moyennement  content | 2  Mécontent | 1  Très mécontent |
| --- | --- | --- | --- | --- | --- | --- |
| 2 | Êtes-vous content(e) des primes et indemnités que vous recevez ? | 5  Très content | 4  Content | 3 Moyennement  content | 2  Mécontent | 1  Très mécontent |
| 3 | Êtes-vous content(e) de la couverture de vos besoins par votre salaire ? | 5  Très content | 4  Content | 3 Moyennement  content | 2  Mécontent | 1  Très mécontent |
| 4 | Êtes-vous content(e) du montant de votre salaire par rapport à vos compétences? | 5  Très content | 4  Content | 3 Moyennement  content | 2  Mécontent | 1  Très mécontent |
| 5 | Êtes-vous content(e) du montant de votre salaire par rapport au volume de votre travail? | 5  Très content | 4  Content | 3 Moyennement  content | 2  Mécontent | 1  Très mécontent |

**2 Questions sur les outils et le cadre de travail**

| 6 | Êtes-vous content(e) de la disponibilité du sang pour les transfusions ? | 5  Très content | 4  Content | 3 Moyennement  content | 2  Mécontent | 1  Très mécontent |
| --- | --- | --- | --- | --- | --- | --- |
| 7 | Êtes-vous content(e) des médicaments dont vous disposez pour faire votre travail ? | 5  Très content | 4  Content | 3 Moyennement  content | 2  Mécontent | 1  Très mécontent |
| 8 | Êtes-vous content(e) des consommables (par ex : coton, alcool…) dont vous disposez pour faire votre travail ? | 5  Très content | 4  Content | 3 Moyennement  content | 2  Mécontent | 1  Très mécontent |
| 9 | Êtes-vous content(e) de la protection contre les risques professionnels (par ex, contre l’exposition au VIH, contre d’autres risques) ? | 5  Très content | 4  Content | 3 Moyennement  content | 2  Mécontent | 1  Très mécontent |
| 10 | Êtes-vous content(e) des imprimés dont vous disposez pour faire votre travail? | 5  Très content | 4  Content | 3 Moyennement  content | 2  Mécontent | 1  Très mécontent |

**3 Questions sur la charge de** travail

| 11 | Êtes-vous content(e) de vos horaires de travail ? | 5  Très content | 4  Content | 3 Moyennement  content | 2  Mécontent | 1  Très mécontent |
| --- | --- | --- | --- | --- | --- | --- |
| 12 | Êtes-vous content(e) de votre charge de travail (quantité de travail, débordé ou pas) ? | 5  Très content | 4  Content | 3 Moyennement  content | 2  Mécontent | 1  Très mécontent |
| 13 | Êtes-vous content(e) de la répartition de la charge de travail entre les membres de votre équipe ? | 5  Très content | 4  Content | 3 Moyennement  content | 2  Mécontent | 1  Très mécontent |
| 14 | Êtes-vous content(e) de la répartition de votre temps de travail entre les soins et vos autres tâches? | 5  Très content | 4  Content | 3 Moyennement  content | 2  Mécontent | 1  Très mécontent |
| 15 | Êtes-vous content(e) du soutien apporté par les autres membres du l’équipe dans l’accomplissement de votre tache? | 5  Très content | 4  Content | 3 Moyennement  content | 2  Mécontent | 1  Très mécontent |

4 Questions sur la tâche

| 16 | Êtes-vous content(e) de la variété de vos tâches (tâches de différentes sortes) ? | 5  Très content | 4  Content | 3 Moyennement  content | 2  Mécontent | 1  Très mécontent |
| --- | --- | --- | --- | --- | --- | --- |
| 17 | Êtes-vous content(e) de l’adéquation qui existe entre vos tâches et vos compétences (est-ce que vos tâches correspondent bien à vos compétences) ? | 5  Très content | 4  Content | 3 Moyennement  content | 2  Mécontent | 1  Très mécontent |
| 18 | Êtes-vous content(e) du niveau de responsabilité professionnelle qui vous est confié ? | 5  Très content | 4  Content | 3 Moyennement  content | 2  Mécontent | 1  Très mécontent |
| 19 | Êtes-vous content(e) de la description qui a été faite de votre tâche? | 5  Très content | 4  Content | 3 Moyennement  content | 2  Mécontent | 1  Très mécontent |
| 20 | Êtes-vous content(e) de la description qui a été faite de votre tâche et ce que vous faite réellement? | 5  Très content | 4  Content | 3 Moyennement  content | 2  Mécontent | 1  Très mécontent |

5 Questions sur l’entente au travail

| 21 | Êtes-vous content(e) de l’entente qui existe entre les membres de votre service ? | 5  Très content | 4  Content | 3 Moyennement  content | 2  Mécontent | 1  Très mécontent |
| --- | --- | --- | --- | --- | --- | --- |
| 22 | Êtes-vous content(e) de la reconnaissance de la qualité de votre travail par vos ***collègues*** ? | 5  Très content | 4  Content | 3 Moyennement  content | 2  Mécontent | 1  Très mécontent |
| 23 | Êtes-vous content(e) de la reconnaissance de la qualité de votre travail par vos ***supérieurs hiérarchiques*** ? | 5  Très content | 4  Content | 3 Moyennement  content | 2  Mécontent | 1  Très mécontent |
| 24 | Êtes-vous content(e) de la façon dont sont faites les notations pour l’avancement de grade? | 5  Très content | 4  Content | 3 Moyennement  content | 2  Mécontent | 1  Très mécontent |
| 25 | Êtes-vous content(e) du respect avec lequel vos supérieurs vous traitent ? | 5  Très content | 4  Content | 3 Moyennement  content | 2  Mécontent | 1  Très mécontent |

6 Questions sur la formation continue

| 26 | Êtes-vous content(e) de la formation que vous ***continuez*** à recevoir ? | 5  Très content | 4  Content | 3 Moyennement  content | 2  Mécontent | 1  Très mécontent |
| --- | --- | --- | --- | --- | --- | --- |
| 27 | Êtes-vous content(e) de la façon dont on  sélectionne les membres du service pour participer aux activités de formation ? | 5  Très content | 4  Content | 3 Moyennement  content | 2  Mécontent | 1  Très mécontent |
| 28 | Êtes-vous content(e) de l’adéquation entre la formation proposée et vos besoins? | 5  Très content | 4  Content | 3 Moyennement  content | 2  Mécontent | 1  Très mécontent |
| 29 | Êtes-vous content(e) de la façon dont les connaissances acquises dans la formation sont utilisées dans votre travail? | 5  Très content | 4  Content | 3 Moyennement  content | 2  Mécontent | 1  Très mécontent |
| 30 | Êtes-vous content(e) des compétences nouvellement acquises lors des formations? | 5  Très content | 4  Content | 3 Moyennement  content | 2  Mécontent | 1  Très mécontent |

7 Questions sur le management

| 31 | Êtes-vous content(e) de l’application des sanctions ***positives*** dans votre service ***en général*** ? | 5  Très content | 4  Content | 3 Moyennement  content | 2  Mécontent | 1  Très mécontent |
| --- | --- | --- | --- | --- | --- | --- |
| 32 | Êtes-vous content(e) des possibilités de participer à la prise de décisions pour résoudre les problèmes d’organisation du travail ? | 5  Très content | 4  Content | 3 Moyennement  content | 2  Mécontent | 1  Très mécontent |
| 33 | Êtes-vous content(e) des informations qu’on vous donne sur la vie de votre ***service***? | 5  Très content | 4  Content | 3 Moyennement  content | 2  Mécontent | 1  Très mécontent |
| 34 | Êtes-vous content(e) des informations qu’on vous donne sur la vie de votre ***établissement*** (les problèmes, les activités, les décisions, la gestion financière…) ? | 5  Très content | 4  Content | 3 Moyennement  content | 2  Mécontent | 1  Très mécontent |
| 35 | Êtes-vous content de la transparence dans la gestion des ressources financières de la structure ? | 5  Très content | 4  Content | 3 Moyennement  content | 2  Mécontent | 1  Très mécontent |

8 Questions sur la satisfaction morale

| 36 | Êtes-vous content(e) de l’issue des accouchements dans votre service (santé des femmes et des nouveau-nés) ? | 5  Très content | 4  Content | 3 Moyennement  content | 2  Mécontent | 1  Très mécontent |
| --- | --- | --- | --- | --- | --- | --- |
| 37 | Êtes-vous content(e) de la qualité de votre propre travail ? | 5  Très content | 4  Content | 3 Moyennement  content | 2  Mécontent | 1  Très mécontent |
| 38 | Êtes-vous content(e) sur le plan religieux de l’aide (service) que vous apportez aux patientes? | 5  Très content | 4  Content | 3 Moyennement  content | 2  Mécontent | 1  Très mécontent |
| 39 | Êtes-vous content(e) que votre lieu de travail ne vous empêche pas de pratiquer votre religion? | 5  Très content | 4  Content | 3 Moyennement  content | 2  Mécontent | 1  Très mécontent |
| 40 | Êtes-vous content(e) de l’image que l’on a de votre profession ? | 5  Très content | 4  Content | 3 Moyennement  content | 2  Mécontent | 1  Très mécontent |

9 Questions sur la stabilité

| 41 | Êtes-vous content(e) de la régularité de versement de votre salaire (salaire versé à temps, en retard…) ? | 5  Très content | 4  Content | 3 Moyennement  content | 2  Mécontent | 1  Très mécontent |
| --- | --- | --- | --- | --- | --- | --- |
| 42 | Êtes-vous content(e) de votre stabilité d’emploi (certitude ou incertitude sur le fait de garder / perdre votre emploi) ? | 5  Très content | 4  Content | 3 Moyennement  content | 2  Mécontent | 1  Très mécontent |
| 43 | Êtes-vous content(e) de la régularité de versement de vos primes? | 5  Très content | 4  Content | 3 Moyennement  content | 2  Mécontent | 1  Très mécontent |
| 44 | Êtes-vous content(e) de votre type de statut (fonctionnaire ou contractuel)? | 5  Très content | 4  Content | 3 Moyennement  content | 2  Mécontent | 1  Très mécontent |

1. Dire le nombre de personnes pour qui vous faites des dépenses **régulières** et **assez importantes.** [↑](#footnote-ref-2)
