# Supplementary material for: Developing a tool to measure satisfaction among health professionals in sub-Saharan Africa
Source: Hum Resour Health. 2013 Jul 4;11:30. doi: 10.1186/1478-4491-11-30 (PMC3704923; doi:10.1186/1478-4491-11-30)
Supplement: Additional file 5 — Items (mean, standard deviation, median)*. [file 1478-4491-11-30-S5.docx]

**Additional file 5: Items (mean, standard deviation, median)***

| **Dimensions** | **Items** | **Sub sample 1** | | | **Sub sample 2** | | | Total | | |
| --- | --- | --- | --- | --- | --- | --- | --- | --- | --- | --- |
|  |  | ^Mean^ | ^SD^ | ^Median^ | ^Mean^ | *^SD^* | ^Median^ | ^Mean^ | ^SD^ | ^Median^ |
| **^F1 Continuing^** | ^(Q30) Skills acquired^ | ^3.46^ | ^1.04^ | ^4.00^ | ^3.35^ | *^1.06^* | ^4.00^ | ^3.40^ | ^1.05^ | ^4.00^ |
| **^education^** | ^(Q28) Relevance^ | ^3.09^ | ^0.97^ | ^3.00^ | ^3.01^ | *^0.99^* | ^3.00^ | ^3.05^ | ^0.98^ | ^3.00^ |
|  | ^(Q29) Skills utilization^ | ^3.21^ | ^1.02^ | ^3.00^ | ^3.12^ | *^1.00^* | ^3.00^ | ^3.17^ | ^1.01^ | ^3.00^ |
|  | ^(Q26) Continuing education you still receive^ | ^2.98^ | ^1.10^ | ^3.00^ | ^2.96^ | *^1.13^* | ^3.00^ | ^2.97^ | ^1.11^ | ^3.00^ |
|  | ^(Q27) Selection for training^ | ^2.82^ | ^1.08^ | ^3.00^ | ^2.73^ | *^1.02^* | ^3.00^ | ^2.77^ | ^1.05^ | ^3.00^ |
| **^F2 Tasks^** | ^(Q19) Job description^ | ^3.68^ | ^0.73^ | ^4.00^ | ^3.69^ | *^0.70^* | ^4.00^ | ^3.69^ | ^0.72^ | ^4.00^ |
|  | ^(Q20) Job description and effective tasks^ | ^3.61^ | ^0.76^ | ^4.00^ | ^3.62^ | *^0.76^* | ^4.00^ | ^3.61^ | ^0.76^ | ^4.00^ |
|  | ^(Q18) Level of responsibility^ | ^3.81^ | ^0.71^ | ^4.00^ | ^3.74^ | *^0.75^* | ^4.00^ | ^3.77^ | ^0.73^ | ^4.00^ |
| **^F3 Management^** | ^(Q34) Information about your institution^ | ^2.83^ | ^0.93^ | ^3.00^ | ^2.79^ | *^0.97^* | ^3.00^ | ^2.81^ | ^0.95^ | ^3.00^ |
| **^style^** | ^(Q33) Information about your department^ | ^3.26^ | ^0.87^ | ^3.00^ | ^3.17^ | *^0.98^* | ^3.00^ | ^3.22^ | ^0.93^ | ^3.00^ |
|  | ^(Q32) Participation in decision making^ | ^3.12^ | ^1.01^ | ^3.00^ | ^3.07^ | *^1.01^* | ^3.00^ | ^3.10^ | ^1.01^ | ^3.00^ |
| **^F4 Salary and^** | ^(Q4) Level of salary and workload^ | ^2.38^ | ^0.88^ | ^2.00^ | ^2.34^ | *^0.85^* | ^2.00^ | ^2.36^ | ^0.86^ | ^2.00^ |
| **^benefits^** | ^(Q1) Level of salary^ | ^2.71^ | ^0.82^ | ^3.00^ | ^2.67^ | *^0.83^* | ^3.00^ | ^2.69^ | ^0.82^ | ^3.00^ |
|  | ^(Q3) Salary and needs^ | ^2.53^ | ^0.78^ | ^3.00^ | ^2.56^ | *^0.79^* | ^3.00^ | ^2.55^ | ^0.78^ | ^3.00^ |
| **^F5 Work^** | ^(Q8) Availability of equipment and materials^ | ^3.19^ | ^0.88^ | ^3.00^ | ^3.05^ | *^0.97^* | ^3.00^ | ^3.12^ | ^0.93^ | ^3.00^ |
| **^environment^** | ^(Q7) Availability of medicines^ | ^3.09^ | ^0.98^ | ^3.00^ | ^3.05^ | *^1.01^* | ^3.00^ | ^3.07^ | ^0.99^ | ^3.00^ |
| **^F6 Moral^** | ^(Q37) Quality of your work^ | ^4.07^ | ^0.62^ | ^4.00^ | ^4.12^ | *^0.62^* | ^4.00^ | ^4.09^ | ^0.62^ | ^4.00^ |
| **^satisfaction^** | ^(Q38) Support to patients from a religious point of view^ | ^4.15^ | ^0.52^ | ^4.00^ | ^4.17^ | *^0.54^* | ^4.00^ | ^4.16^ | ^0.53^ | ^4.00^ |
| **^F7 Workload^** | ^(Q12) Workload^ | ^3.17^ | ^0.92^ | ^3.00^ | ^3.16^ | *^0.88^* | ^3.00^ | ^3.17^ | ^0.90^ | ^3.00^ |
|  | ^(Q11) Work schedule^ | ^3.43^ | ^0.87^ | ^4.00^ | ^3.39^ | *^0.84^* | ^4.00^ | ^3.41^ | ^0.86^ | ^4.00^ |
|  | ^(Q14) Balance between care and other activities^ | ^3.41^ | ^0.77^ | ^4.00^ | ^3.38^ | *^0.74^* | ^3.00^ | ^3.40^ | ^0.75^ | ^3.00^ |
| **^F8 Job stability^** | ^(Q42) Concern about losing your job^ | ^3.94^ | ^0.74^ | ^4.00^ | ^3.92^ | *^0.76^* | ^4.00^ | ^3.93^ | ^0.75^ | ^4.00^ |
|  | ^(Q41) Salary paid on time^ | ^3.93^ | ^0.92^ | ^4.00^ | ^3.89^ | *^0.99^* | ^4.00^ | ^3.91^ | ^0.96^ | ^4.00^ |
|  | ^(Q44) Status (civil servant. tenure track. contract)^ | ^3.75^ | ^0.97^ | ^4.00^ | ^3.69^ | *^1.00^* | ^4.00^ | ^3.72^ | ^0.98^ | ^4.00^ |

* Scores range from 1 (very dissatisfied) to 5 (very satisfied).
