# Supplementary material for: Developing a tool to measure satisfaction among health professionals in sub-Saharan Africa
Source: Hum Resour Health. 2013 Jul 4;11:30. doi: 10.1186/1478-4491-11-30 (PMC3704923; doi:10.1186/1478-4491-11-30)
Supplement: Additional file 6 — Component matrix. [file 1478-4491-11-30-S6.docx]

**Additional file 6: Component matrix**

|  | | | | | | | | |  |
| --- | --- | --- | --- | --- | --- | --- | --- | --- | --- |
|  | Component | | | | | | | |  |
|  | 1 | 2 | 3 | 4 | 5 | 6 | 7 | 8 |  |
| Q1. Level of salary | .038 | .059 | .856 | .068 | .075 | .047 | .030 | .025 |  |
| Q3. Salary and needs | .039 | .074 | .827 | .020 | .026 | .077 | .122 | -.049 |  |
| Q4. Level of salary and workload | .098 | .060 | .819 | .115 | .090 | .045 | .046 | .053 |  |
| Q7. Availability of medicines | .121 | .031 | .085 | .175 | .095 | .066 | .831 | .064 |  |
| Q8. Availability of equipment and materials | .071 | .140 | .106 | .073 | .061 | .012 | .856 | .060 |  |
| Q11. Work schedule | .095 | .085 | -.019 | .815 | .113 | .117 | .065 | .039 |  |
| Q12. Workload | .085 | .141 | .085 | .858 | .058 | .035 | .065 | .097 |  |
| Q14. Balance between care and other activities | .102 | .176 | .161 | .709 | .091 | .067 | .144 | .039 |  |
| Q18. Level of responsibility | .140 | .746 | .023 | .035 | .173 | .087 | .097 | .077 |  |
| Q19. Job description | .159 | .856 | .111 | .179 | .090 | .000 | .032 | .138 |  |
| Q20. Job description and effective tasks | .189 | .825 | .098 | .255 | .127 | -.024 | .076 | .074 |  |
| Q26. Continuing education you receive | .779 | .128 | .124 | .135 | .159 | .007 | .121 | .092 |  |
| Q27. Selection for training | .722 | .187 | .047 | -.075 | .349 | -.002 | .103 | -.074 |  |
| Q28. Relevance (Continuing education) | .856 | .126 | .053 | .122 | .095 | .087 | .001 | .029 |  |
| Q29. Skills utilisation and continuing education | .810 | .121 | .032 | .125 | .155 | .068 | .024 | .081 |  |
| Q30. Skills acquired and continuing education | .851 | .039 | -.002 | .044 | .045 | .189 | .055 | .147 |  |
| Q32. Participation in decision making | .227 | .232 | .031 | .016 | .751 | .118 | .016 | -.009 |  |
| Q33. Information about your department | .165 | .138 | .089 | .083 | .835 | .021 | .027 | .065 |  |
| Q34. Information about your institution | .215 | .024 | .103 | .227 | .721 | .054 | .157 | .102 |  |
| Q37. Quality of your work | .085 | .204 | .104 | .092 | .056 | .073 | .088 | .829 |  |
| Q38. Support to patients on a religious point of view | .120 | .049 | -.070 | .066 | .064 | .088 | .038 | .851 |  |
| Q41. Salary paid on time | .081 | .044 | .006 | .094 | .056 | .807 | .103 | -.023 |  |
| Q42. Concern about losing your job | .080 | .061 | .006 | .003 | -.009 | .843 | .020 | .115 |  |
| Q44. Status (civil servant. tenure track. contract) | .097 | -.041 | .168 | .107 | .113 | .726 | -.046 | .075 |  |
| Extraction method : principal component analysis. Varimax rotation with Kaiser normalization. | | | | | | | | | |
